# Nuclear envelope-associated endosomes deliver surface proteins to the nucleus

**DOI:** 10.1038/ncomms9218

**Published:** 2015-09-10

**Authors:** Alexandre Chaumet, Graham D. Wright, Sze Hwee Seet, Keit Min Tham, Natalia V. Gounko, Frederic Bard

**Affiliations:** 1 Institute of Molecular and Cell Biology, 61 Biopolis Drive, Proteos, Singapore 138673, Singapore; 2 Institute of Medical Biology, 8A Biomedical Grove, #06-06 Immunos, Singapore 138648, Singapore; 3 Joint IMB-IMCB Electron Microscopy Suite, 20 Biopolis Street, #B2-14 Matrix, Singapore 138671, Singapore; 4 Department of Biochemistry, National University of Singapore, 21 Lower Kent Ridge Road, Singapore 119077, Singapore

## Abstract

Endocytosis directs molecular cargo along three main routes: recycling to the cell surface, transport to the Golgi apparatus or degradation in endolysosomes. Pseudomonas exotoxin A (PE) is a bacterial protein that typically traffics to the Golgi and then the endoplasmic reticulum before translocating to the cytosol. Here we show that a substantial fraction of internalized PE is also located in nuclear envelope-associated endosomes (NAE), which display limited mobility, exhibit a propensity to undergo fusion and readily discharge their contents into the nuclear envelope. Electron microscopy and protein trapping in the nucleus indicate that NAE mediate PE transfer into the nucleoplasm. RNAi screening further revealed that NAE-mediated transfer depends on the nuclear envelope proteins SUN1 and SUN2, as well as the Sec61 translocon complex. These data reveal a novel endosomal route from the cell surface to the nucleoplasm that facilitates the accumulation of extracellular and cell surface proteins in the nucleus.

## Abstract (web summary)

Endocytosis typically directs proteins on a recycling route back to the plasma membrane, transport to the Golgi apparatus or delivery to the lysosome. Here Chaumet *et al.* describe a population of vesicles that can fuse directly with the outer nuclear membrane and deliver cargo into the nuclear envelope, where it can be translocated into the nucleoplasm.

## Graphical abstract

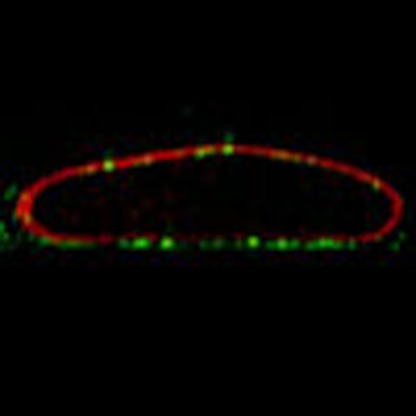

The eukaryotic nucleus is bounded by two lipid bilayer membranes that join to form pores that mediate traffic of soluble proteins both into and out of the nuclear space^1^. Extracellular proteins and surface receptors can also traffic into the nucleus, where they can exert potent effects on gene expression and alter cell function^2^,^3^,^4^,^5^,^6^,^7^. Transport of these key extracellular factors into the nucleus is therefore a critical regulator of cellular responses to external cues, but the mechanisms that transfer them into the nucleoplasm remain poorly defined.

Following endocytosis, extracellular proteins can be directed to the recycling endosomes, endolysosomes or Golgi apparatus. The bacterial protein Pseudomonas exotoxin A (PE) binds to the cell surface receptor LRP1 for internalization before trafficking from endosomes to the Golgi, then transport to the endoplasmic reticulum (ER) where it is translocated to the cytosol^8^. We previously determined that PE trafficking from endosomes to the Golgi is dependent on the SNARE protein STX16 (refs 9, 10). Transfer to the ER is dependent on the KDEL-R and ERGIC2 proteins, while translocation to the cytosol is mediated by the Sec61 translocon^11^. Cell surface receptors have been proposed to follow a similar route, although it has not been clearly established and a requirement for trafficking through the Golgi has yet to be demonstrated.

Here we describe a fourth endosomal route that transports cell surface receptors to the nucleoplasm through docking and membrane fusion of a population of endosomes with the nuclear envelope.

## Results

### PE vesicles are closely associated with the nuclear envelope

**Figure 1:**
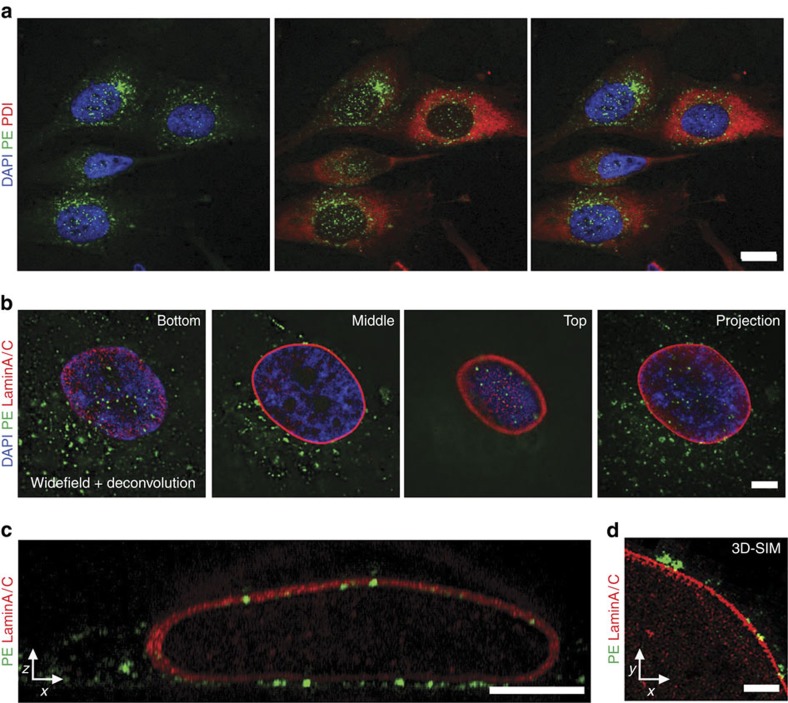
PE-containing vesicles are closely associated with the nuclear envelope. (**a**) Confocal imaging of PE+ vesicular structures in MG63 cells stained with the ER marker PDI and the DNA intercalating dye 4,6-diamidino-2-phenylindole (scale bar, 25 μm). Image is representative of 10 independent experiments. (**b**) Deconvolution microscopy image demonstrating close association of PE+ structures with the nuclear envelope as revealed by fluorescent staining of the envelope components lamin A and C (scale bar, 5 μm). (**c**) Z/X reconstruction image demonstrating close association of PE+ structures with the lamin sheet (scale bar, 5 μm). (**d**) 3D-SIM super-resolution microscopy image of PE and lamin A/C (scale bar, 1 μm). Image is representative of three independent experiments.

While tracking the transport of fluorescently labelled PE in the osteosarcoma cell line MG63, we not only detected the typical Golgi localization associated with this protein but also observed a recurrent and consistent association of toxin-labelled vesicular structures with the nucleus (Fig. 1a and Supplementary Fig. 1a). By using deconvolution microscopy and three-dimensional (3D) modelling together with ‘super-resolution' structured illumination microscopy (SIM), we observed that the PE+ vesicular structures were located either at the top or bottom of the nucleus in close association with the nuclear envelope and lamin sheet (Fig. 1b–d and Supplementary Movie 1). The ER marker protein disulfide isomerase (PDI) was excluded from the areas above and below the nucleus, suggesting that the space between nuclear envelope and cell membrane is limited. By extension, it suggests that an active mechanism is driving the accumulation of these PE-laden vesicles in this location (Fig. 1a).

### Nucleus-associated PE vesicles do not require Golgi traffic

**Figure 2:**
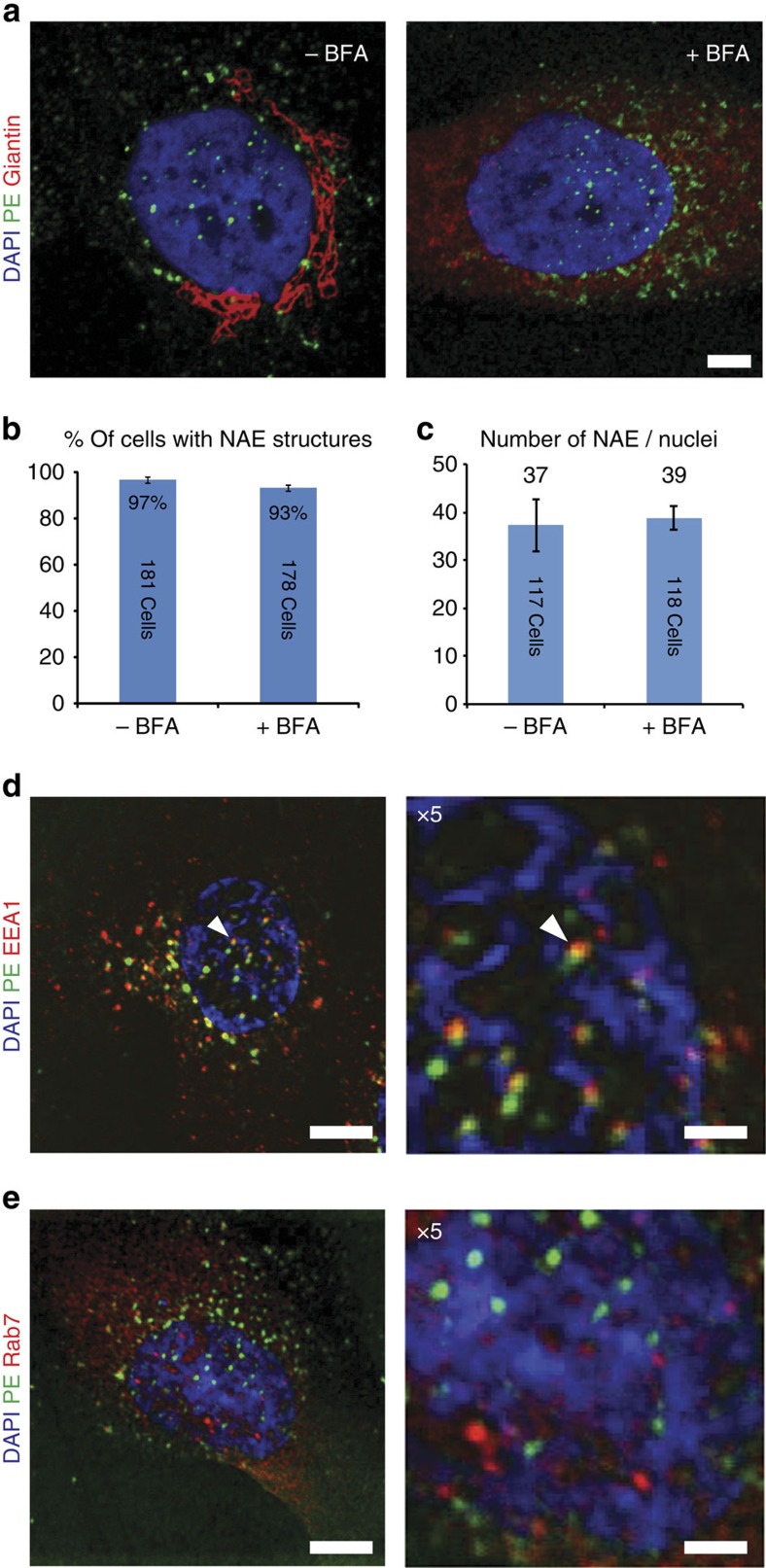
Vesicles associated with the nuclear envelope are derived from early endosomes. (**a**) Cells pre-treated with Brefeldin A (BFA) or dimethylsulfoxide (DMSO) both before and during incubation with Alexa 488-PE were stained for giantin (scale bar, 5 μm). (**b**) Percentage of cells displaying NAE structures. Representative of three independent experiments (error bars indicate s.d.). (**c**) Average number of NAE structures by nuclei. Representative of three independent experiments (error bars indicate s.d.). (**d**) Co-labelling of PE with early endosomal marker EEA1. (**e**) Co-labelling with the late endosomal marker Rab7 (scale bar, 10 μm, × 5 image: scale bar, 2 μm). Data shown in **c** and **d** are from three experiments.

Nuclear envelope-associated vesicles containing fluorescent PE could be detected as early as 15 min after protein addition to MG63 cells, although staining intensity was relatively weak at this early time point. After 1 h PE exposure, toxin-laden vesicles were clearly visible in association with the nuclear envelope in the majority of cells, and with only trace staining in the Golgi area, suggesting that toxin accumulation did not involve transit via the Golgi apparatus. Accordingly, cells pre-treated with the Golgi blocker Brefeldin A for 30 min before toxin exposure still displayed numerous PE+ vesicles associated with the nuclear envelope (Fig. 2a–c).

Further analysis revealed that PE+ vesicles partially co-localized with the early endosome antigen 1 (EEA1; Fig. 2d). In contrast, there was no co-localization of PE with the late endosome marker Rab7, lysosomal marker Lamp1 or secretory pathway marker ERGIC53 (Fig. 2e and Supplementary Fig. 2a,b). Taken together, these data strongly suggested that the PE-laden vesicles detected in association with the nuclear envelope are derived from early endosomes (hereafter referred to as nuclear envelope-associated endosomes (NAE)).

### NAE display fusion events and progressive loss of content

**Figure 3:**
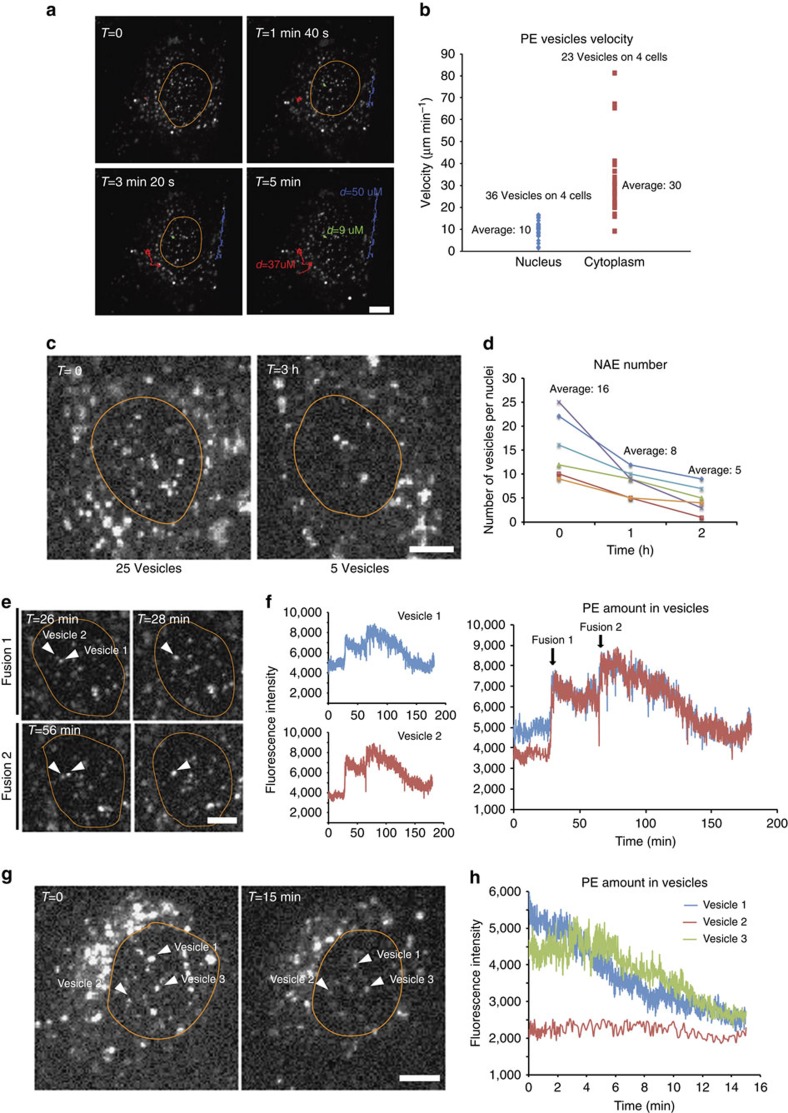
NAE display low motility, frequent fusion events and progressive loss of vesicular content. (**a**) MG63 cells were incubated for 1 h with Alexa 488-conjugated PE and then imaged every 0.5 s for a total duration of 5 min. (**b**) Quantification of vesicle velocity. (**c**) NAE number over time in an individual live cell. (**d**) NAE frequency in six individual cells. (**e**) Apparent NAE fusion event. (**f**) Increase in PE fluorescence intensity consistent with NAE fusion. Images were taken every 5 s for a total duration of 3 h. (**g**,**h**) Decreasing fluorescence intensity of PE-laden NAEs over time suggesting progressive loss of vesicular contents (scale bar, 10 μm). Images and data are representative of six independent experiments.

PE-positive NAE appeared within 10 min of incubation with PE and disappeared in a few hours: after a PE pulse of 1 h, the nuclear-associated PE signal decreased rapidly over the next 3 h (Supplementary Fig. 2c). Using live-cell imaging, we observed that NAE were largely static and exhibited an average velocity at least threefold lower than that of their cytoplasmic counterparts (Fig. 3a,b and Supplementary Movie 2). We also detected a limited number of nucleus-associated vesicles that rapidly trafficked over the nucleus before abruptly stopping and becoming immobile, suggesting that NAE are not spatially constrained but are instead docked on the nuclear envelope. We next monitored PE-exposed cells using images captured every 5 s for a total duration of 3 h after addition of toxin, during which time there was a marked decrease in the number of NAE detected per nucleus, despite the fact that these structures could not be observed to exit the nuclear area (Fig. 3c,d). Subsequent analyses revealed that NAE moving independently would frequently make contact with one another and become indistinguishable thereafter, accompanied by an increase in fluorescence intensity to a level roughly equivalent to the sum of the parent vesicles, strongly suggesting that NAE can readily undergo fusion events (Fig. 3e,f and Supplementary Movie 3).

Individual NAE frequently displayed a progressive decrease in PE fluorescence signal while some adjacent NAE exhibited stable fluorescence signals throughout the observation period, suggesting selective loss of vesicular cargo rather than a general photobleaching effect (Fig. 3h, Supplementary Fig. 3a,b and Supplementary Movie 4). The rate of PE fluorescence decrease was uniform and comparable between NAE (Fig. 3h), and often lasted tens of minutes up to 1 h (Fig. 3f and Supplementary Fig. 3a,b). In an analysis of 35 different NAE, 22 displayed a clear decrease in fluorescence signal (Supplementary Fig. 3a,b). Since these vesicles appeared to be docked on the nuclear envelope, we postulated that NAE can undergo partial fusion with the envelope and progressively discharge their content into the nuclear envelope. Indeed, partial fusion of endosomes with the plasma membrane has been recently reported by other investigators^12^. We next sought to determine whether NAE co-localize with nuclear pores markers; however, we were unable to detect any co-localization (Supplementary Fig. 2d).

### NAE content is translocated to the nucleoplasm

**Figure 4:**
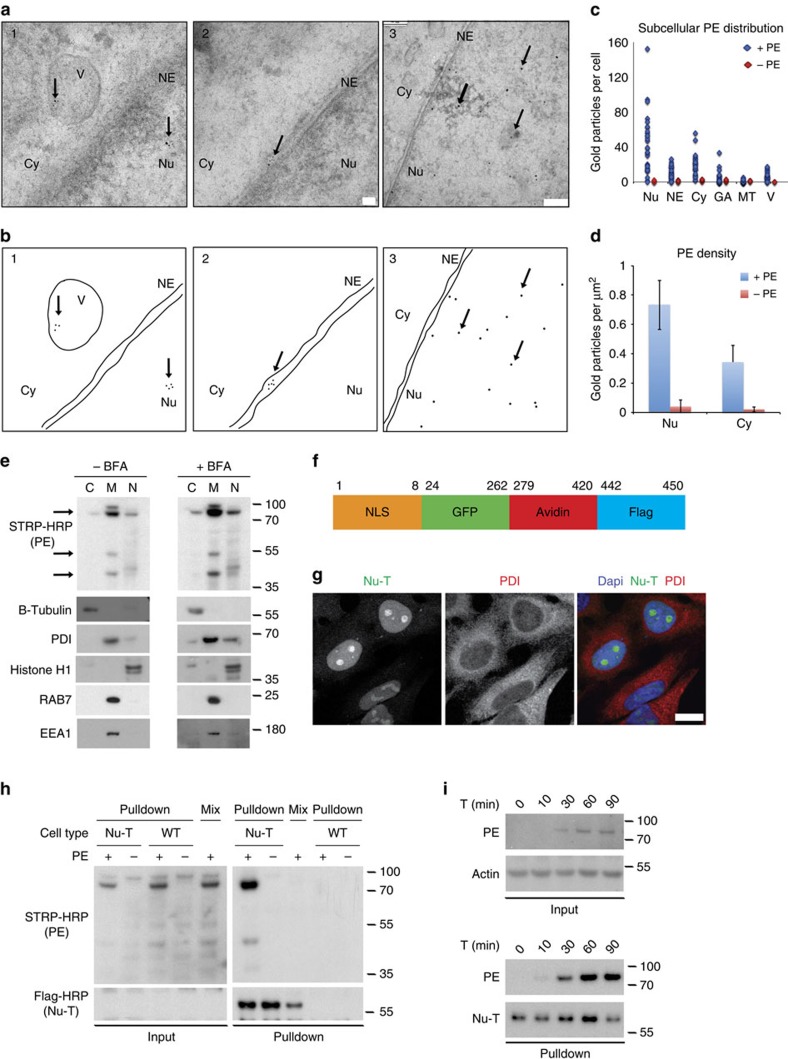
PE localizes in the nuclear envelope lumen and nucleoplasm. (**a**) MG63 cells intoxicated for 1 h with biotinylated PE and stained with gold-labelled streptavidin (scale bar, 100 nm). (**b**) Schematic of image shown in **a** with gold particles indicated by arrows. (**c**) Number of gold particles per cell in different compartments. (**d**) Number of gold particles per μm^2^ in the cytosol and nucleus. Representative of two independent experiments (error bar indicates s.d.). (**e**) Fractionation of cells intoxicated with biotinylated PE for 1 h with (right panel) or without (left panel) BFA pre-treatment (C, cytosolic fraction; M, membrane fraction; N, nuclear fraction). (**f**,**g**) Nu-T construct and its nuclear localization (scale bar, 25 μm). (**h**) Biotinylated PE detection after pulldown of Nu-T (Supplementary Fig. 5a,b). (**i**) Kinetics of PE pulldown. (**e**,**h**,**i**) Representative of at least five independent experiments. Cy, cytosol; GA, Golgi apparatus; NE, nuclear envelope; Nu, nucleus; MT, mitochondria; V, vesicles.

We next proceeded to assess NAE by electron microscopy (EM), which identified these structures as being in close proximity to and occasionally merging with the nuclear envelope membranes (Fig. 4a–c and Supplementary Fig. 4c). Furthermore, PE could be detected in the lumen of the nuclear envelope and was also present on the nucleoplasmic side of the membrane (Fig. 4a–c and Supplementary Fig. 4a–d). This nucleoplasmic PE staining was even more pronounced when visualized using ultra-small gold particles with silver enhancement labelling. Quantification indicated that PE staining density was about twice as high in nucleoplasm than in the cytosol, consistent with the idea that PE translocates directly from NAE to the nucleoplasm (Fig. 4d). Together, these observations suggested that PE+ NAE fuse with the nuclear envelope to transfer their content into the nucleus.

To confirm our EM results, we next incubated MG63 cells with biotinylated PE for 1 h then performed subcellular fractionation experiments to separate nuclei from the cellular membranes and cytosolic fractions. Using this approach, we were able to confirm that although the majority of PE partitioned with the membrane fraction, a significant pool of protein could also be detected in the nuclear fraction. To exclude the possibility that contamination with NAE contributed to PE detection in the nuclear fraction, we next generated a HeLa cell line that stably expressed a chimeric avidin sequence featuring a nuclear localization signal as well as Green Fluorescent Protein (GFP) and a FLAG tag (hereafter referred to as nuclear trap (Nu-T); Fig. 4f,g). Biotinylated PE could be readily detected interacting with Nu-T in the nucleus of HeLa cells just 1 h after protein exposure (Fig. 4h), but not in wild-type (WT) cells incubated with biotinylated PE and mixed with Nu-T immediately before cell lysis and immunoprecipitation (Supplementary Fig. 6b). Pre-treatment with the Golgi blocker Brefeldin A was unable to abolish the nuclear localization of PE, further indicating that trafficking of this protein to the nucleus is independent of the Golgi apparatus (Fig. 4e and Supplementary Fig. 6c). To evaluate the kinetics of PE transfer to the nucleus, we next lysed Nu-T cells at different time points after exposure to PE. A faint nuclear signal could be detected as soon as 10 min after PE addition, followed by a progressive increase over the following 80 min (Fig. 4i). Together, these data suggest that PE translocation to the nucleus commences soon after the first vesicles are detected near the envelope and does not require transit through the Golgi.

### PE nuclear translocation is SUN1/2 and Sec61 dependent

**Figure 5:**
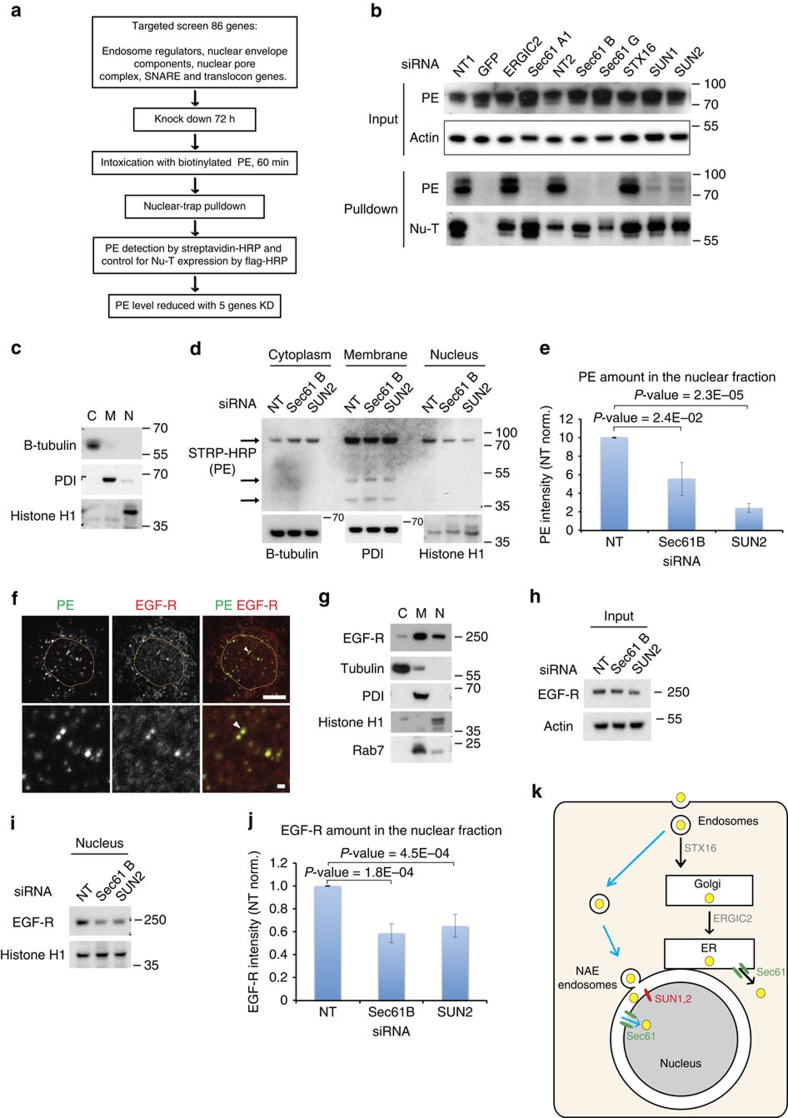
PE requires SUN1/2 and the translocon complex for transport to the nucleoplasm. (**a**) Flowchart and (**b**) results of siRNA screening; PE and Nu-T detection as in Fig. 4. Representative of five different replicates. (**c**–**e**) MG63 cell fractionation after Sec61B or SUN2 knockdown. (**c**) Fractionation controls (as in Fig. 4). (**d**) PE subcellular distribution in MG63 cells after Sec61B and SUN2 knockdown. Representative of three independent repeats. (**e**) Quantification of PE in nuclear fraction after Sec61B and SUN2 knockdown. Error bars at s.d. *t*-Test type 2, two tails. (**f**) Co-labelling of PE with EGFR. Nucleus is delimited by yellow circle. Scale bar, 10 μm. (**g**) A431 cells fractionation. (**h**) Input loading control of A431 protein extract. (**i**) EGFR nuclear distribution in A431 cells after Sec61B and SUN2 knockdown. Representative of three independent repeats. (**j**) Quantification of EGFR in nuclear fraction. Error bars at s.d. *t*-Test type 2, two tails. (**k**) Two intracellular routes of cargos. After endocytosis, NAE dock on the nucleus, discharging their content into the nucleoplasm in a SUN1/2- and Sec61 translocon-dependent manner.

We next used Nu-T cells together with a targeted RNA interference screening approach to identify the genes that mediated nuclear translocation of PE, including proteins involved in nuclear envelope regulation, as well as components of the translocon complex (Fig. 5a and Supplementary Fig. 5). The complete list of 24 genes screened in these experiments is provided in Supplementary Table 1. Depletion efficiency was not tested for all target proteins (see Supplementary Fig. 6d for expression levels of a subset of target proteins). STX16 and ERGIC2, which are known to be required for PE intoxication in HeLa cells, were used as negative controls (Fig. 5b)^10^. In contrast, depletion of the nuclear envelope proteins SUN1 and SUN2 potently impaired PE translocation to the nucleus (Fig. 5b), and this effect could be successfully replicated using three or more different short interfering RNAs (siRNAs) for each protein (Supplementary Fig. 6f), without adversely affecting the nuclear localization of Nu-T (Supplementary Fig. 6g).

The SUN1 and SUN2 proteins are embedded in the inner membrane of the nuclear envelope and protrude into the lumen, while also interacting with Nesprin proteins in the outer membrane to form a bridge between these layers^13^. Knockdown of the four Nesprin genes described to date, either alone or in combination, was unable to substantially restrict PE translocation to the nucleus^14^ (Supplementary Fig. 6h). However, since the individual depletion of Nesprin 3 and Nesprin 4, as well as several double knockdowns induced toxicity, it is not possible to exclude a role for these molecules in nuclear import of PE.

Other hits were three genes that encode the heterotrimeric Sec61 translocon complex, which forms a channel in membranes. Depletion of each of the Sec61 subunits, alpha1 (A1), beta (B) and gamma (G), resulted in a similar drastic reduction of PE translocation to the nucleus (Fig. 5b). We confirmed these results by using several different siRNAs against Sec61B subunits, each of which replicated the restriction of PE translocation to the nucleus (Supplementary Fig. 6i), while preserving localization of Nu-T (Supplementary Fig. 6j). Using super-resolution microscopy, we could detect Sec61 in the nuclear envelope of MG63 cells (Supplementary Fig. 6k). Furthermore, a recent report has shown that a fraction of Sec61 is localized at the inner nuclear membrane^15^. Taken together, these data strongly suggest that Sec61 mediates PE translocation across the inner nuclear membrane.

We next sought to confirm these results in a system that did not depend on Nu-T system. We therefore performed siRNA knockdown of Sec61B or SUN2 in HeLa cells and MG63 cells before PE exposure, then carried out cellular fractionation as before, and confirmed depletion of Sec61B or SUN2 using western blotting (Supplementary Fig. 6l). Fractionation efficiency was determined by blotting for tubulin, PDI and histone H1 (Fig. 5c). In the absence of either Sec61B or SUN2, the nuclear fraction of PE staining was significantly reduced, whereas the membrane-bound fraction was unaffected (Fig. 5d,e and Supplementary Fig. 6m).

PE binds to the cell surface receptor LRP1 (ref. 8). In MG63 cells, we found LRP1 staining to co-localize with PE in NAE structures and to be significantly enriched in the nucleus (Supplementary Fig. 6a). Furthermore, LRP1 could be detected in a nuclear fraction (Supplementary Fig. 7b). When Sec61B or SUN2 were depleted, this nuclear fraction was significantly reduced (Supplementary Fig. 7c,d), indicating that PE and LRP1 follow the same pathway into the nucleus.

To test whether Epidermal Growth Factor Receptor (EGFR) also accumulates in the nucleus following the same pathway, MG63 cells were incubated with PE for 1 h and stained for EGFR, revealing co-localization in NAE (Fig. 5f). While EGFR nuclear immunofluorescence staining was not as prominent as for LRP1, subcellular fractionation revealed a nuclear fraction (Fig. 5g). This fraction was reduced in cells depleted of Sec61B or SUN2, while total EGFR was not affected (Fig. 5h–j). The reduction was comparable to that for PE in a similar experiment (Fig. 5e).

## Discussion

Overall, our results indicate that PE and other cell surface molecules can follow at least two intracellular routes: one leading to the cytosol by way of the Golgi and ER, and one to the nucleoplasm via NAE (Fig. 5k). The first route appears to require a few hours and is clearly important for inhibition of cytoplasmic ribosomal activity by PE, as demonstrated by the protective effect of ERGIC2 and STX16 depletion^10^. The second route leads to delivery of PE into the nucleoplasm within 1 h. The existence of this second route is further substantiated by the fact that nuclear PE tends to be unprocessed (that is, full length), whereas cytosolic PE is mostly cleaved. Indeed, PE is cleaved by Furin in the Trans Golgi Network (TGN), which promotes its toxic activity^16^. Furthermore, we could not detect any interaction of PE with KPNB1 (importin beta 1), a key factor for transport through the nuclear pore (Supplementary Fig. 7g). This data further support the fact that cytosolic PE is not actively transported into the nucleus. On the other hand, full-size PE is presumably unable to diffuse freely through the nuclear pores due to its size. Therefore, nuclear PE may not be able to inhibit cytoplasmic ribosomes, explaining perhaps why the second route is not very efficient for intoxication.

On the other hand, this trafficking route is probably used by various endogenous proteins such as LRP1 and EGFR, and could explain the nuclear accumulation of various growth factors and receptors previously reported^5^. NAE represent a novel delivery route that permits cell surface proteins and extracellular molecules to access the genome and alter cellular function. The NAE pathway may therefore represent an alternative mechanism by which external stimuli can influence cellular activity independently of the conventional signalling cascades that operate in the cytosol. This route of surface protein transport into the nucleus is likely a tightly regulated event rather than a constitutive process. In addition, this pathway could represent an interesting route for delivery of therapeutic agents directly into the nucleus.

## Methods

### Gene synthesis and cloning

The sequence of the Nu-T NLS-GFP-Avidin-Flag was synthesized and cloned in a pDONR221 vector using gene synthesis (Invitrogen, Thermo Fisher Scientific, Carlsbad, CA). The sequence was then transferred using a pLenti6.3-DEST vector with the Gateway system.

### Cell line culture and stable transduction

All cell lines come from ATCC. Lentiviruses were generated according to the manufacturer's instructions (Invitrogen). WT HeLa cells were transduced to stably express Nu-T. All cell lines (HeLa WT, HeLa Nu-T, MG63 and A431) were maintained in high-glucose Dulbecco's modified Eagle's medium supplemented with 10% fetal calf serum at 37 °C in a 10% CO_2_ incubator. Cells infected with GFP virus were subjected to fluorescence-activated cell sorting sorting to enrich for GFP+ cells before rapid expansion and freezing. All experiments were performed on cells passaged fewer than 10 times after thawing.

### Fluorescent PE uptake

PE (#341215, Merck Millipore, Darmstadt, Germany) was labelled with AnaTag Hilyte Fluor 488 according to the manufacturer's instructions (#72048, AnaSpec, Fremont, CA). Typically, MG63 cells were seeded 2 days before incubation with ∼250 μg ml^−1^ fluorescent PE for 60 min (concentrations as low as 5 μg ml^−1^ produced similar images).

### Immunofluorescence

Cells were seeded onto glass coverslips in 24-well dishes (Thermo Fisher Scientific) and incubated at 37 °C, 10% CO_2_ for 8–16 h. In some experiments, the cells were incubated with 10 μg ml^−1^ Bredfedin A for 30 min both before and during PE intoxication. All subsequent steps were performed at room temperature. Cells were washed with Dulbecco's Phosphate Buffer Saline (D-PBS), fixed for 15 min using 4% paraformaldehyde in D-PBS, washed with D-PBS, then permeabilized and blocked with 0.2% Triton X-100, 2% Foetal Bovine Serum (FBS) in D-PBS for 15 min before antibody staining (EEA1 (#C45B10, 1:300, Cell Signaling Technology, Beverly, MA), EGFR (#2232L, 1:200, Cell Signaling Technology), ERGIC53 (#E1031, 1:200, Sigma-Aldrich, St. Louis, MO), giantin (#ab24586, 1:1,000, Abcam, Cambridge, UK), Lamin a/c (#ab8984, 1/500, Abcam), Lamp1 (#ab24170, 1:300, Abcam), LRP1 (#L2420, 1:200, Sigma-Aldrich), NPC (#ab24609, 1:100, Abcam), PDI (#RL77, 1:500, Abcam), Rab7 (#ab137029, 1:300, Abcam), Sec61B (# ab78276, 1:200, Abcam), SUN1 (#HPA008461, 1:300, Sigma-Aldrich) and SUN2 (# HPA001209, 1:300, Sigma-Aldrich)). Cells were washed three times using 2% Foetal Bovine Serum in D-PBS and then subsequently stained for 45 min with secondary Alexa Fluor-conjugated antibodies and Hoechst (Invitrogen). Cells were mounted onto glass slides using FluorSave (Merck Millipore).

### Scanning confocal microscopy

An Olympus FV1000 upright confocal microscope (Olympus Microscopes, Essex, UK) equipped with 405, 488, 561 and 647 nm lasers for excitation and spectral/band-pass emission filters was used for acquisition of confocal images and construction of z-stacks. An Olympus Plan Apo × 60/1.35 oil immersion objective lens was used. The confocal pinhole was set to 1 Airy unit. For z-stacks, the z-spacing was fixed to meet the Nyquist criteria. Figure preparation was carried out using Fiji software^17^.

### 3D-SIM and wide-field deconvolution

A DeltaVision OMX v4 Blaze microscope (Applied Precision, GE Healthcare, Issaquah, WA), equipped with 488 and 568 nm lasers (3D-SIM), a solid-state illuminator (WF) for excitation, and BGR filter drawer was used (emission wavelengths 436/31 for 4,6-diamidino-2-phenylindole, 528/48 for Alexa 488 and 609/37 for Alexa 568). An Olympus Plan Apochromat × 100/1.4 Point Spread Function (PSF) oil immersion objective lens was used with liquid-cooled Photometrics Evolve EM-CCD cameras (Photometrics, Tucson, AZ) for each channel. Fifteen images per section per channel were acquired (made up of three rotations and five phase movements of the diffraction grating) at a z-spacing of 0.125 μm (refs 18, 19). Structured illumination reconstruction or deconvolution followed by alignment was conducted using the SoftWorX (Applied Precision) programme with figure preparation in Imaris (Andor-Bitplane, Zurich) and Fiji software^17^. Imaris (Andor-Bitplane, Zurich) was used for 3D rendering and to generate the animation. DeltaVision OMX images were directly imported, and serial *XY* and *XZ* planes were produced using the Orthogonal Slicer tool. The 3D modelization was created using the Surpass tool, with maximum display intensity for Lamin and the Spots tool (intensity and size threshold) to enable visualization of PE.

### Spinning disk live-cell confocal microscopy

Live-cell imaging was performed using a motorized Nikon Ti inverted microscope (Nikon, Tokyo, Japan) equipped with a Plan Apo × 60/1.4 oil immersion objective lens, a 491-nm laser (FRAP-3D laser launch; Photometrics) and CSU-22 scanning head (Yokogawa Electric Corp., Tokyo, Japan), Evolve EM-CCD camera (Photometrics) and the Nikon Perfect Focus System. Cells were maintained at 37 °C in a 5% CO_2_ humidified environment using an on-stage incubator (Chamlide, Live Cell Instrument, Seoul, South Korea). Time-lapse imaging was controlled using MetaMorph software (100 ms exposure, EM-gain 100, 15% laser power, Molecular Devices, LLC, Sunnyvale, CA). The image analysis was completed using Fiji software (ImageJ, National Institutes of Health (NIH), Bethesda, MD).

### Electron microscopy

MG63 cells were grown on coverslips and exposed to 750 μg ml^−1^ biotinylated PE for 60 min before fixation with 4% paraformaldehyde, 0.25% glutaraldehyde in 0.1 M phosphate buffer, pH 7.4 for 1 day, and then stored at 4 °C. The coverslips were rinsed in ice-cold 0.1 M phosphate buffer (pH 7.4) and then in 0.1 M sodium cacodylate trihydrate (pH 7.4). After fixation, the samples were exposed to osmium fixative solution (1% OsO_4_+1.5% K_3_Fe(CN)_6_ in 0.1 M sodium cacodylate trihydrate, pH 7.4) for 1 h at room temperature. Samples were then washed in dH_2_O and dehydrated through an ascending ethanol series. Infiltration was continued using Epon 812 resin in 100% ethanol and then fresh Epon 812 resin overnight. Finally, the samples were embedded in Epon 812 fresh resin and polymerized at 56 °C for 24 h. Ultrathin sections (∼70 nm) were cut on an ultra-microtome with a diamond knife (Diatome Inc., Bern, Switzerland) and carefully positioned on 100 mesh Nickel grids (Electron Microscopy Systems, Hatfield, PA). The sections were supported by formvar and carbon.

Immunostaining was performed following a protocol modified from Hearn *et al.*^20^. Grids with sections (with or without biotinylated PE) were incubated with 1% periodic acid for 10 min and then rinsed in dH_2_O followed by PBS (pH 7.4). The grids were pre-incubated on a drop of 0.05 M PBS (pH 7.4), with 0.15% glycine, 0.2% Cold Water Fish Gelatin (CWFG) and 1% bovine serum albumin for 1 h and then placed on a drop of 6-nm gold-streptavidin antibody (1:50) or ultra-small gold-streptavidin antibody (1:50) in the same solution without bovine serum albumin overnight at 4 °C (Electron Microscopy Systems). The following day, the grids were washed in PBS and incubated with 1% glutaraldehyde in dH_2_O, followed by a wash with dH_2_O. Finally, ultra-small gold labelling was subjected to silver enhancement using GoldEnhance EM kits according to the manufacturer's instructions (Nanoprobes, Yaphank, NY). As an additional control, some grids of cells (with or without exposure to biotinylated PE) were incubated in the same way but without the antibody.

Ultrathin sections were stained for 5 min in 5% uranylacetate in dH_2_O followed by Reynolds lead citrate for 2 min and examined under a JEM-2200FS (Jeol USA, Peabody, MA) at 100 kV. Immunogold staining was considered adequate in the absence of labelling inside mitochondria. All controls were negative for immunogold labelling.

### Cell fractionation

Cells were seeded at the desired densities in six-well dishes (Thermo Fisher Scientific) and incubated at 37 °C, 10% CO_2_ for 8–16 h. PE toxin or Epidermal Growth Factor (EGF) biotinylation were performed using a DSB-X Biotin Protein labelling Kit (#D-20655, Molecular Probes, Thermo Fisher Scientific), according to the manufacturer's protocol. Cells were incubated for 60 min with PE at a final concentration of 5 μg ml^−1^ for HeLa cells and 3 μg ml^−1^ for MG63 cells. A431 cells were stimulated 30 min with EGF 50 ng ml^−1^. In some cases, the cells were incubated with 10 μg ml^−1^ Bredfedin A for 30 min both before and during PE intoxication. Cells were fractionated with Cell Surface Protein Isolation Kit (#89881, Thermo Fisher Scientific) according to the manufacturer's protocol. Membranes were incubated with actin (#3280, 1:5,000, Abcam), β-tubulin (#ab6046, 1:5,000 Abcam), EEA1 (#E7659, 1:1,000, Sigma-Aldrich), EGFR (#2232L, 1:1,000, Cell Signaling Technology), histone H1 (#sc-10806, 1:1,000, Santa Cruz), PDI (#RL77, 1:15,000, Abcam), LRP1 (#L2420, 1:1,000, Sigma-Aldrich), Rab7 (#ab137029, 1:1,000, Abcam), Sec61B (#ab78276, 1:5,000, Abcam), streptavidin-horseradish peroxidase (HRP; #21130, 1:10,000, Thermo Fisher Scientific) and SUN2 (# HPA001209, 1:2,000, Sigma-Aldrich). The western blot quantification was done using Fiji software package (ImageJ, NIH).

### Nu-T pulldown assay

Cells were seeded at the desired densities in six-well dishes (Thermo Fisher Scientific) and incubated at 37 °C, 10% CO_2_ for 16 h. Biotinylated PE toxin was then added to HeLa NLS_GFP_Avidin_Flag or HeLa WT cells for 60 min at a final concentration of 5 μg ml^−1^. Cells were incubated with 10 μg ml^−1^ Bredfedin A for 30 min both before and during PE intoxication. Cells were then washed and lysed for 30 min in 500 μl lysis buffer (Tris 50 mM (pH 8.0), 170 mM NaCl and 0.5% NP-40, together with 7.5 mM biotin to prevent post-lysis interactions) and complete protease inhibitor (Roche Applied Science, Mannheim, Germany) before being centrifuged for 30 min at 16,000*g* and 4 °C. The supernatant was incubated overnight with 15 μl anti-FLAG M2 Magnetic Beads (#m8823, Sigma-Aldrich). After three washes, the beads were suspended in 40 μl of *×* 2 SDS loading buffer (#161-0737, Bio-Rad, Hercules, CA) supplemented with β-mercaptoethanol, followed by boiling at 95 °C for 5 min. Samples were resolved by SDS–polyacrylamide gel electrophoresis using bis-tris 4–12% NuPage gels as per the manufacturer's instructions (#NP036BOX, Life Technologies, Thermo Fisher Scientific). The samples were then transferred onto nitrocellulose membranes and blocked using 3% milk dissolved in Tris Buffered Salin Tween (TBS-T) (50 mM Tris (pH 8.0, 4 °C), 150 mM NaCl and 0.1% Tween-20) for 15 min at room temperature. Membranes were incubated with streptavidin-HRP (#21130, 1:10,000, Thermo Fisher Scientific), Flag-HRP (#a8592, 1:10,000, Sigma-Aldrich) or actin (#3280, 1:5,000, Abcam). For actin antibody staining, membranes were washed with TBS-T and then incubated with secondary HRP-conjugated antibodies (GE Healthcare). Membranes were then washed with TBS-T before ECL exposure. Uncropped western blot are presented in Supplementary Fig. 8.

### Gene knockdown

Knockdown assays were performed in six-well dishes (Thermo Fisher Scientific). Reverse transfection was performed using 3 μl of 50 μM siRNA (Thermo Fisher Scientific) per well, together with 180 μl Opti-MEM (Invitrogen) and 20 μl HiPerFect (#301707, Qiagen, Hilden, Germany). After incubation for 20 min to allow complex formation, 300,000 HeLa NLS_GFP_Avidin_Flag cells, MG63 cells or A431 cells were added to each well in a final volume of 3 ml for a 50 nM siRNA. The cells were then incubated for 72–96 h at 37 °C in a 10% CO_2_ incubator.

## Additional information

**How to cite this article:** Chaumet, A. *et al.* Nuclear envelope-associated endosomes deliver surface proteins to the nucleus. *Nat. Commun.* 6:8218 doi: 10.1038/ncomms9218 (2015).

## Supplementary Material

Supplementary Information Supplementary Figures 1-8 and Supplementary Table 1

Supplementary Movie 1 Z-stack and its orthogonal view of confocal images of PElabeled structures and Lamin A/C staining, plus a 3D rendering of nucleus-associated vesicles containing PE.

Supplementary Movie 2 Live imaging of Alexa Fluor 488-PE loaded cells every 0.5 sec over a period of 5 min. Three vesicles are tracked in green (nucleus), red and blue (cytoplasm).

Supplementary Movie 3 Live imaging of Alexa Fluor 488-PE loaded cells every 5 sec over three hours. Movie corresponds to Figure 3C, E.

Supplementary Movie 4 Live imaging of Alexa Fluor 488-PE loaded cells every 0.5 sec over 15 minutes. Movie corresponds to Figure 3G.
