# Supplementary material for: Nuclear envelope-associated endosomes deliver surface proteins to the nucleus
Source: Nat Commun. 2015 Sep 10;6:8218. doi: 10.1038/ncomms9218 (PMC4579783; doi:10.1038/ncomms9218)
Supplement: Supplementary Information — Supplementary Figures 1-8 and Supplementary Table 1 [file ncomms9218-s1.pdf]

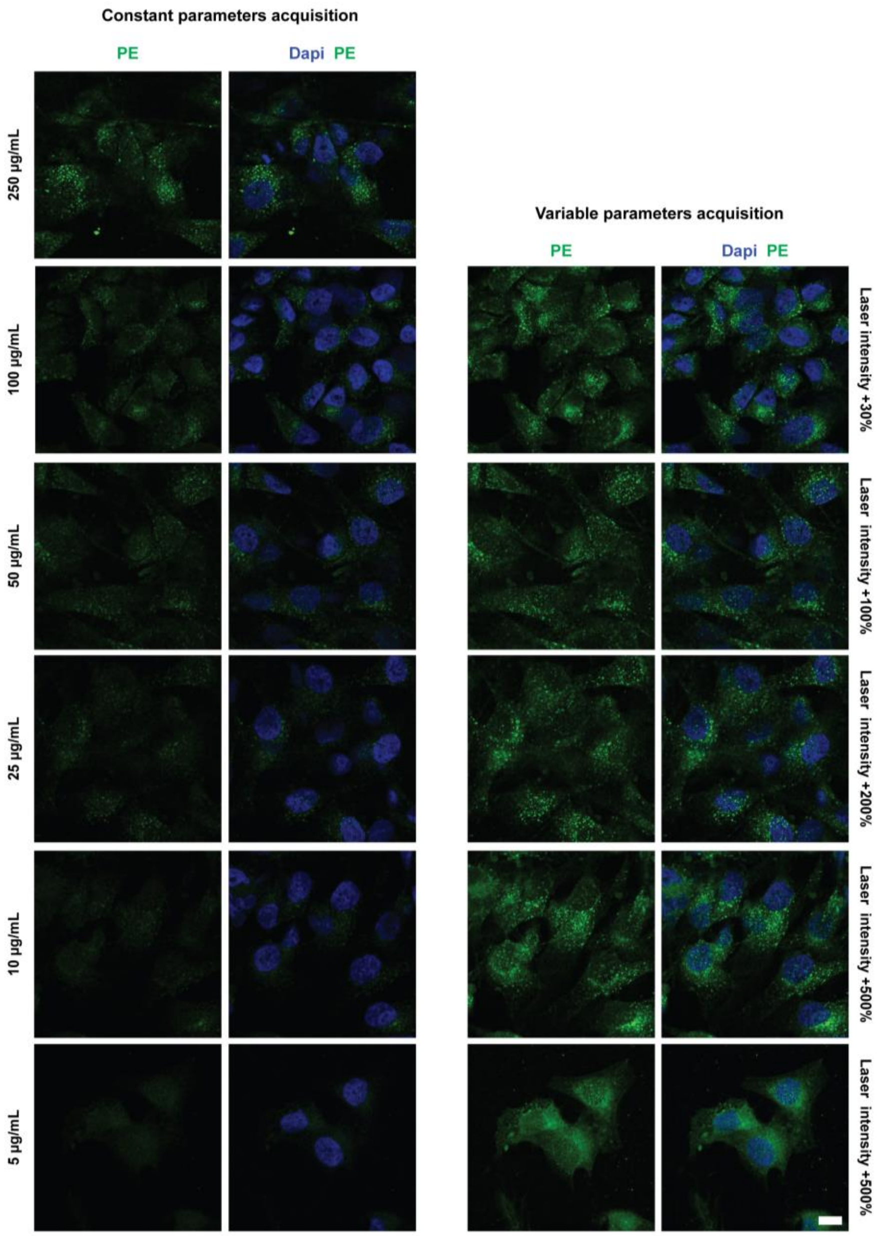

Supplemental Figure 1: MG63 cells were intoxicated with increasing concentration of PE for 1 hour. Image acquisition was done either at constant parameters acquisition (Left panels) or with increasing laser intensity to reach an equivalent signal (Right panels). Scale 25 µm.

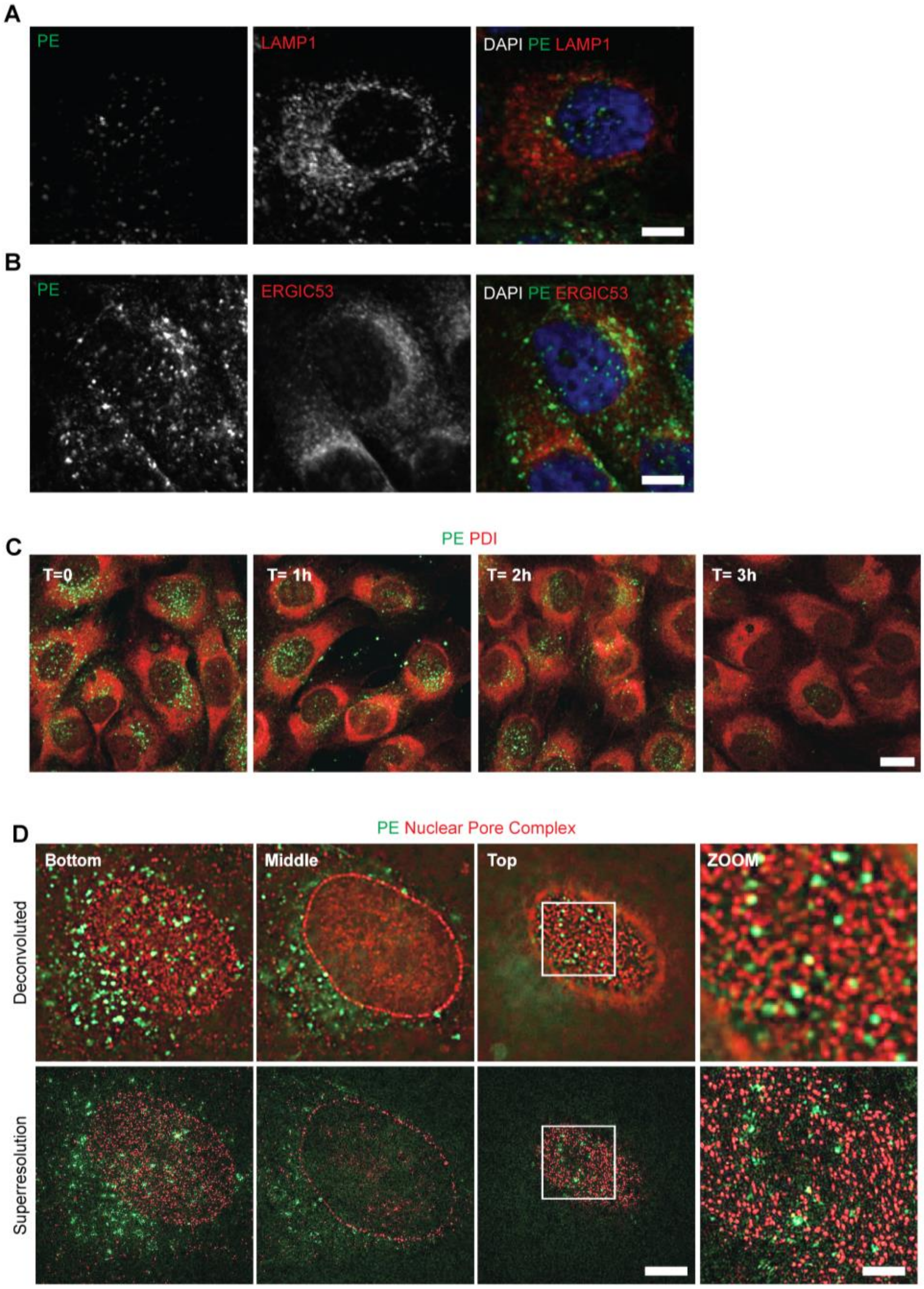

Supplementary Figure 2: MG63 cells incubated with Alexa Fluor 488-PE (green) and stained with (A) LAMP1 antibody (red) or (B) ERGIC53 antibody (red). Images are representative of two independent experiments. Scale, 10 µm (C) Cells were intoxicated for 1 h with Alexa Fluor 488-PE and fixed after washing (T= 0) or at 1 h, 2 h or 3 h later, then labeled with anti-PDI antibody (red). Series is representative of four independent experiments. Scale, 25 µm (D) Representative images of two independent experiments of deconvolution microscopy (Top) and 3D SIM super-resolution microscopy (bottom) of MG63 cells incubated with Alexa Fluor 488-PE and stained for Nuclear Pore Complex. Scale, 5 µm.

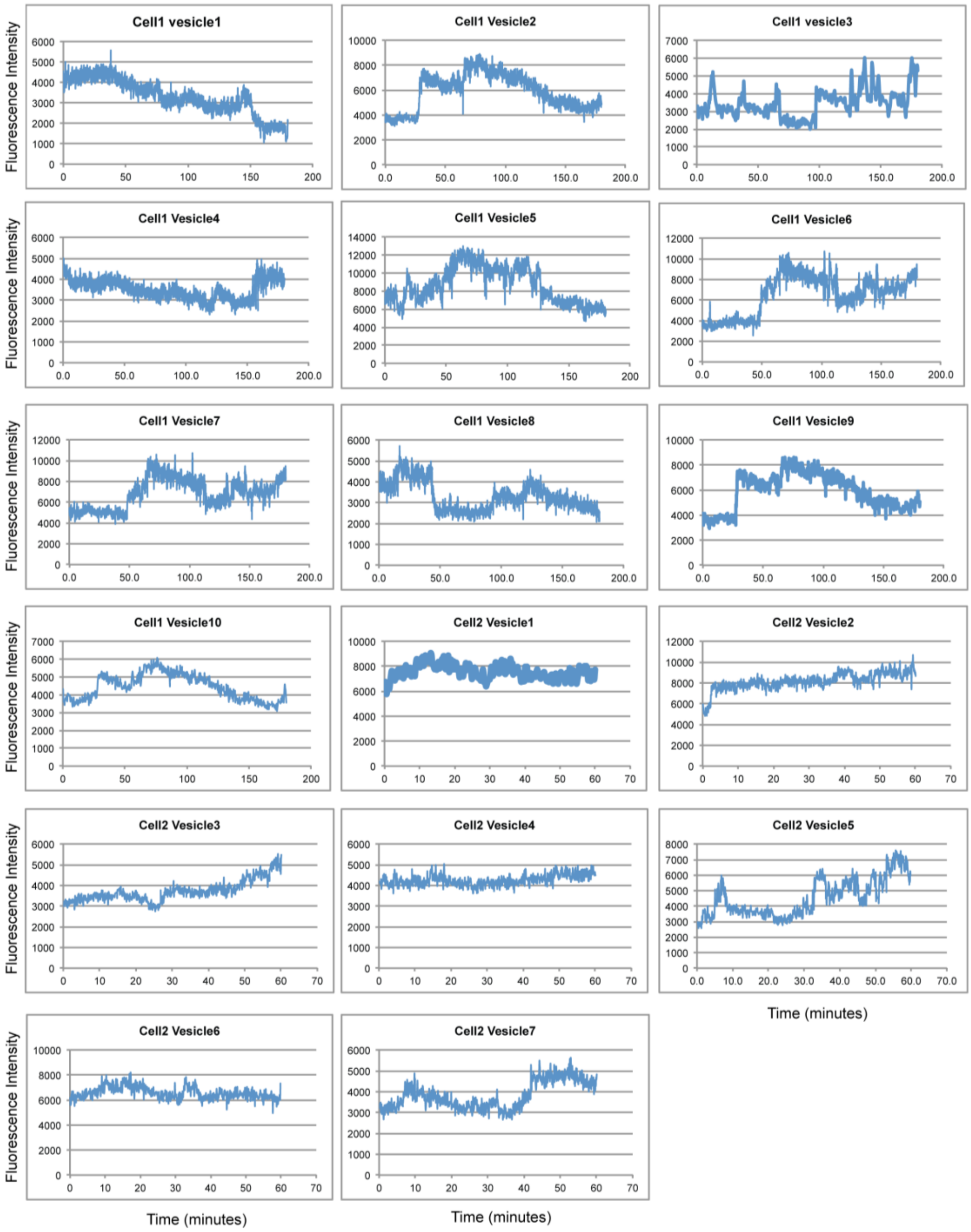
Cell1 vesicle1
Cell1 Vesicle2
Cell1 vesicle3
Cell1 Vesicle4
Cell1 Vesicle5
Cell1 Vesicle6
Cell1 Vesicle7
Cell1 Vesicle8
Cell1 Vesicle9
Cell1 Vesicle10
Cell2 Vesicle1
Cell2 Vesicle2
Cell2 Vesicle3
Cell2 Vesicle4
Cell2 Vesicle5
Cell2 Vesicle6
Cell2 Vesicle7
Fluorescence Intensity
Time (minutes)

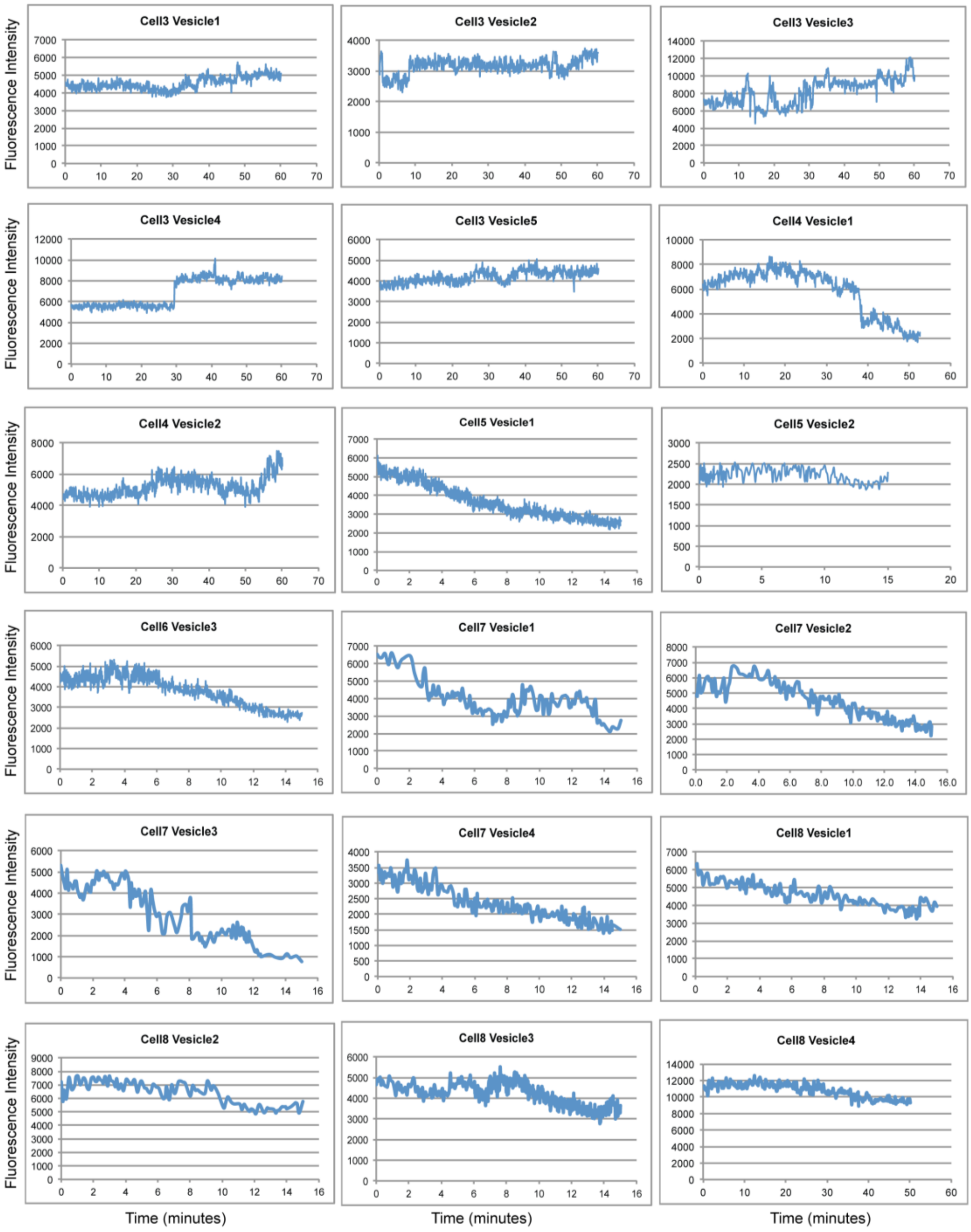

Supplementary Figure 3: (A, B) Quantification of the fluorescence of individual nuclear envelope-associated (NEA) endosome over time in different cells, as indicated in each graph.

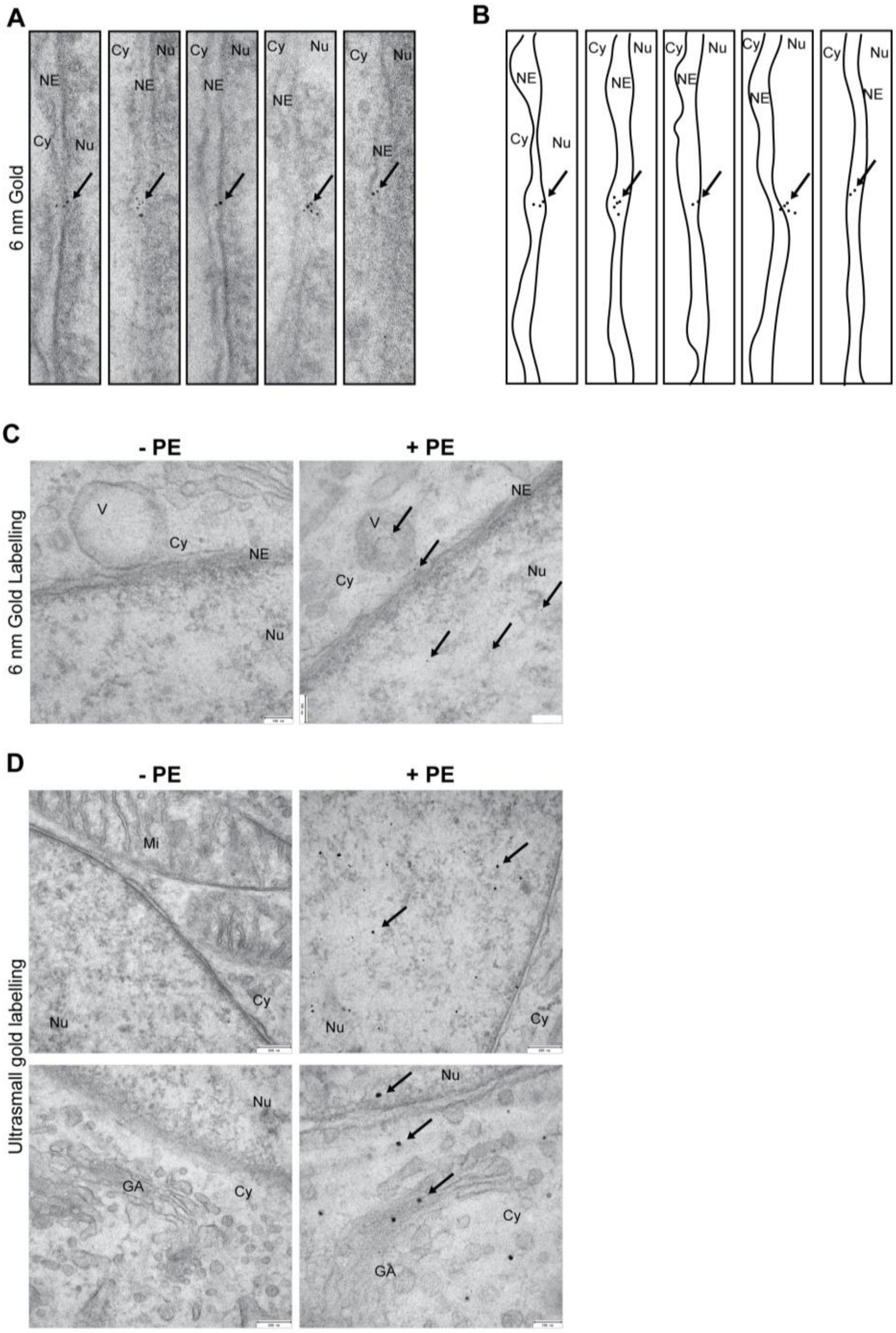

Supplementary Figure 4: (A,B) MG63 cells intoxicated for 1 h with or without biotinylated-PE and stained with gold-labelled streptavidin. (C) MG63 cells intoxicated for 1 h with or without biotinylated-PE and stained with ultra-small gold-labelled streptavidin. Scale, 100 nm (D) Examples of EM images of PE localization in the lumen of the nuclear envelope.

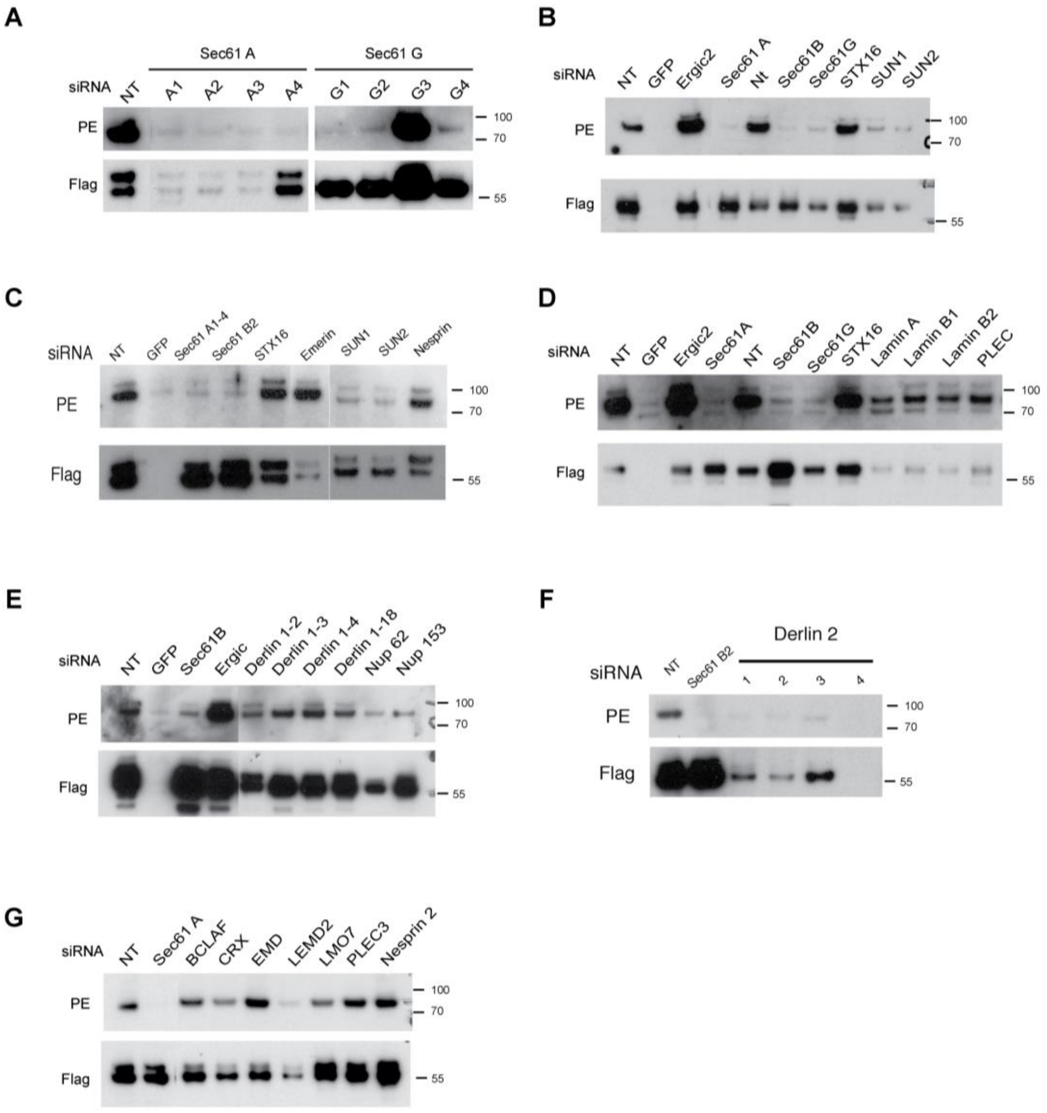

Supplementary Figure 5: Examples of the Nuclear Trap (Nu-T) pull-down assays. Screening results for the various genes tested, as indicated in each figure panel. Biotynilated PE is revealed with streptavidin-HRP and Nu-T with Flag-HRP. Genes Knock Down are indicated on the top of each lanes. MW are indicated on the right of each blot.

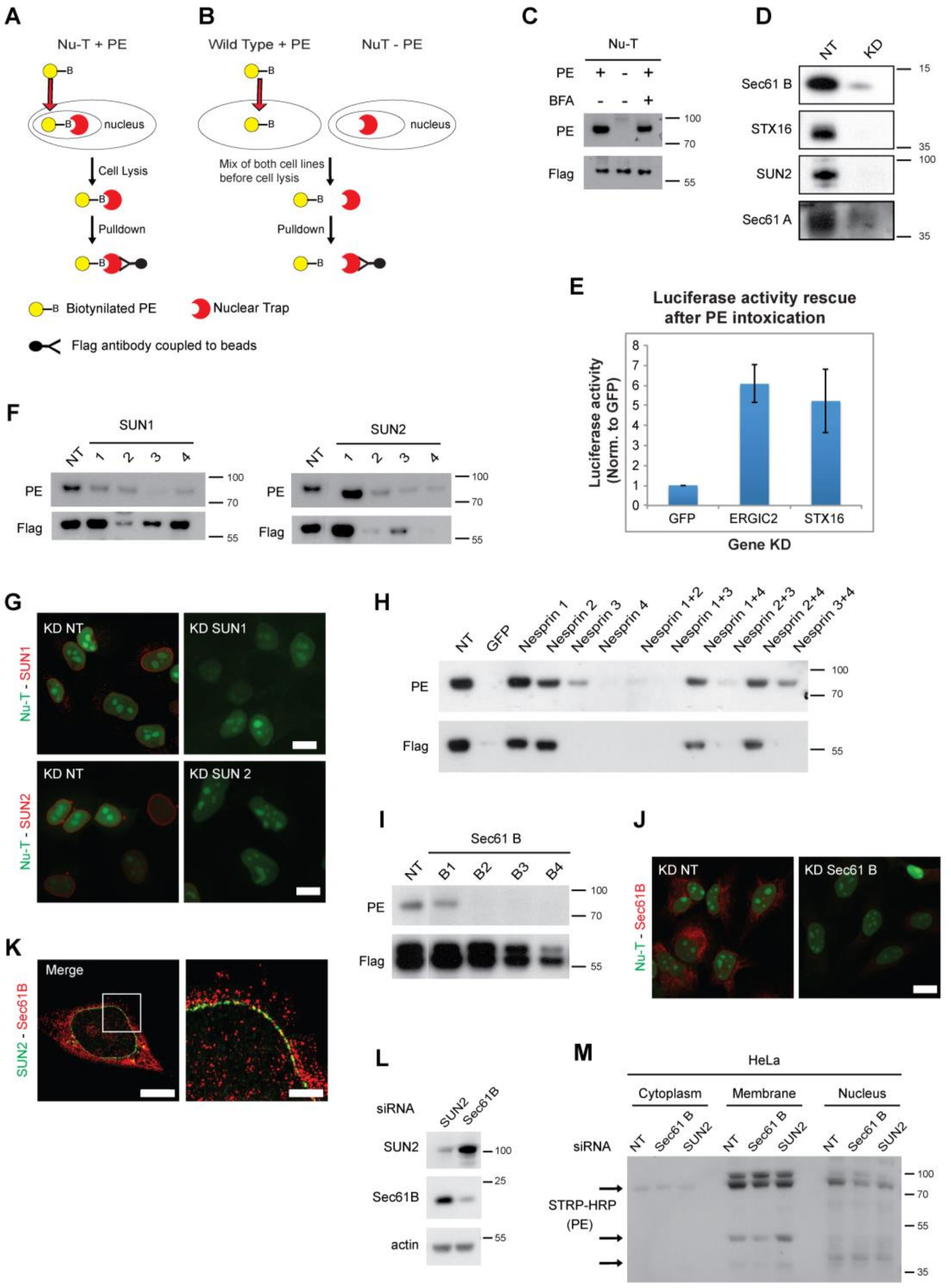

Supplementary Figure 6: (A,B) Schema of PE pull-down for Nu-T cells and control for non-specific binding test: wild-type (WT) cells incubated with PE for 1h and mixed with Nu-T expressing cells before cell lysis and Nu-T immunoprecipitation. (C) Representative result of of 3 independent experiments where Nu-T cells were pre-treated with BFA or DMSO before and during incubation with PE for 1h. (D) Representative western blot for the target proteins Sec61B, STX16, SUN2 and Sec61A before and after treatment with the corresponding siRNA pool. NT, non-targeting; KD, knock down. (E) Average of 3 independent experiments, luciferase activity after PE incubation for 7h. Error bar is s.d. (F) Effects of individual anti-SUN1/2 siRNAs in Nuclear Trap (Nu-T) pull-down assay. (G) Assessment of SUN1/2 knockdown on Nu-T subcellular localization (scale 25 µm) (H) Nu-T pull-down assay after single depletion or co-depletion of Nesprins 1 to 4. (I) Nu-T pull-down

assay after treatment with individual siRNAs against Sec61B. (J) Assessment of Sec61B knockdown on Nu-T subcellular localization (scale 25 µm). (K) Superresolution imaging of Sec61 translocon and nuclear envelope as revealed by SUN2-GFP expression (left scale 10µm, right scale 3 µm). (L) Representative cell fractionation protein depletion evaluation. (M) Representative of 3 independent experiments of subcellular fractionation of HeLa cells after incubation for 1 h with biotinylated PE detected with Streptavidin-HRP.

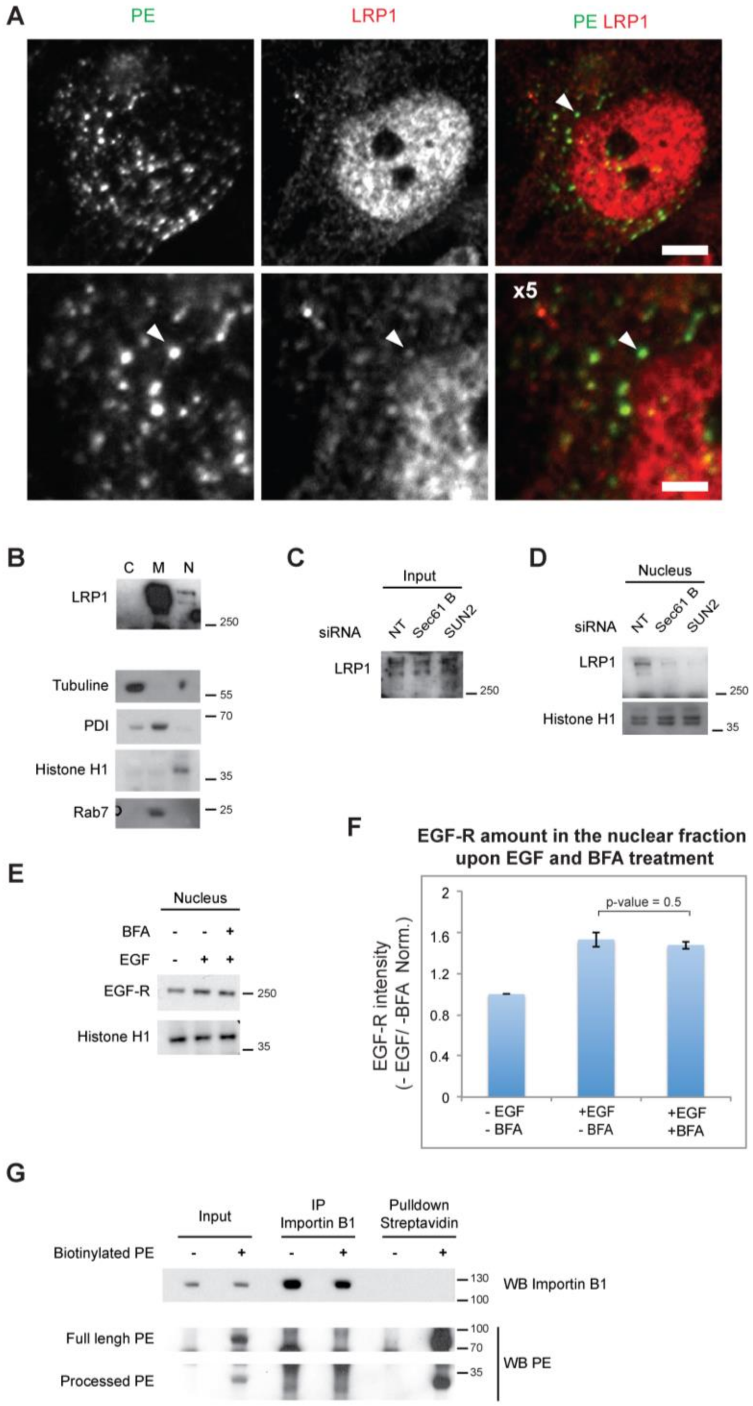

Supplementary Figure 7: (A) Co-labelling of PE with LRP1. Scale, 10 µm, x5: 2 µm. Representative of two independent repeats. (B) MG63 cells fractionation. (C) Input loading control of MG63 protein extract after knock down. (D) LRP1 nuclear distribution in MG63 cells after Sec61B and SUN2 knockdown. Representative of two independent repeats. (E) Nuclear fraction of A431 cells were pre-treated with BFA or DMSO before and during incubation 30 min with EGF. Representative of 3 independent repeats. (F) Quantification of EGF-R in nuclear fraction. Error bars are s.d. T-TEST type 2, 2 tails. (G) Immunoprecipitation of Importin B1 and Streptavidin after intoxication with mock (-) or biotinylated PE (+). Western Blots were revealed with anti Importin B1 and PE antibodies, showing no interactions. IP: Immunoprecipitation, WB: Western Blot.

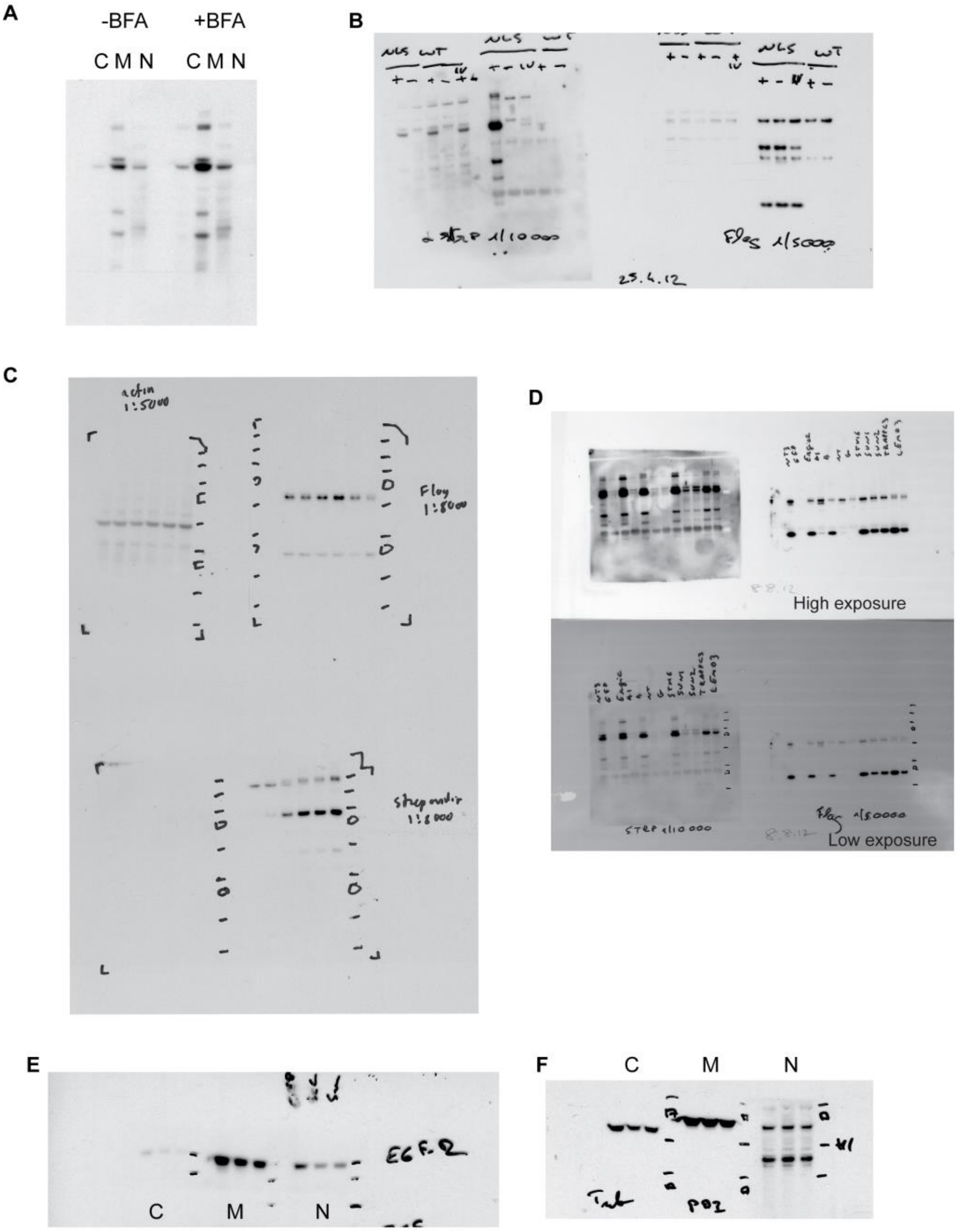

Supplemental Figure 8: (A) uncropped western blot corresponding to Figure 4E. (B) uncropped western blot corresponding to Figure 4H. (C) uncropped western blot corresponding to Figure 4I. (D) uncropped western blot corresponding to Figure 5B. (E,F) uncropped western blot corresponding to Figure 5I. C: cytosolic fraction; M: Membrane fraction; N: Nuclear fraction.

	Gene	Gene id	Alias	Catalogue #
1	Derlin1	79139		D-010733-02
2	Derlin2	51009		D-010576-01
3	Sec61 A1	29927		D-021503-04
4	Sec61 B	10952		D-021504-02
5	Sec61 G	23480		D-012509-01
6	EMD	2010	Emerin	M-011025-01
7	LEMD2	221496		M-017941-00
8	LEMD3	23592	MAN1	M-006306-01
9	LMO7	4008		M-019252-02
10	Syne1	23345	Nesprin1	M014039-02
11	Syne2	23224	Nesprin2	M-019259-01
12	Syne3	161176	Nesprin3	M016637-01
13	Syne4	163183	Nesprin4	M-016874-01
14	PLEC1	5339		M-003945-03
15	LMNA	4000		M-004978-01
16	LMNB1	4001		M-005270-01
17	LMNB2	84823		M-005290-00
18	UNC84A	23353	Sun1	M-025277-01
19	UNC84B	25777	Sun2	M-009959-00
20	BCLAF1	9774		M-020734-01
21	CRX	1406		M-011112-01
22	GFP			P-002048-01
23	Syntaxin 16	NM_001001433		M-019468-01
24	ERGIC2	51290		M-021151-01

Translocon
Nuclear envelope
Controls

Supplementary Table 1: List of the genes targeted in the siRNA screen.
